# Evaluating Metrics Applied to the Medical Science Liaison (MSL) Role: A Survey-Based Study of Canadian MSL Leaders

**DOI:** 10.1007/s43441-021-00291-y

**Published:** 2021-05-04

**Authors:** Munaza Saleem, Lisa Cesario, Lisa Wilcox, Marsha Haynes, Simon Collin, Peter Langlois, Stevie Kenyon, Andrew Chilelli

**Affiliations:** 1 Hoffmann-La Roche Ltd., Mississauga, ON Canada; 2 Pfizer Canada Inc., Kirkland, QC Canada; 3 Janssen Inc., Toronto, ON Canada; 4 AbbVie Corp., Saint-Laurent, QC Canada; 5 AstraZeneca Canada Inc., Mississauga, ON Canada; 6 Placencia Holdings Ltd., Hamilton, ON Canada; 7 Astellas Pharma Canada Inc., Markham, ON Canada

**Keywords:** Survey, Medical Science Liaison, Metrics, Qualitative, MSL, Pharmaceutical

## Abstract

**Introduction:**

Metrics utilized within the Medical Science Liaison (MSL) role are plentiful and traditionally quantitative. We sought to understand the current use and value of metrics applied to the MSL role, including the use of qualitative metrics.

**Methods:**

We developed a list of 70 MSL leaders working in Canada, spanning 29 companies. Invitations were emailed Jun 16, 2020 and the 25-question online survey was open for 3 weeks. Questions were designed to assess demographics as well as how and why metrics are applied to the MSL role. Data analyses were descriptive.

**Results:**

Responses were received from 44 leaders (63%). Of the 42 eligible, 45% had ≤ 2 years of experience as MSL leaders and 86% supported specialty care products over many phases of the product lifecycle. A majority (69%) agreed or strongly agreed that metrics are critical to understanding whether an MSL is delivering value, and 98% had used metrics in the past year. The most common reason to use metrics was ‘to show value/impact of MSLs to leadership’ (66%). The most frequently used metric was ‘number of health-care professional (HCP) interactions’, despite this being seen as having moderate value. Quantitative metrics were used more often than qualitative, although qualitative were more often highly valued.

**Conclusion:**

The data collected show a lack of agreement between the frequency of use for some metrics and their value in demonstrating the contribution of an MSL. Overall, MSL leaders in our study felt qualitative metrics were a better means of showing the true impact of MSLs.

**Supplementary Information:**

The online version contains supplementary material available at 10.1007/s43441-021-00291-y.

## Introduction

The Medical Science Liaison (MSL) and related field-based Medical Affairs (MA) roles represent critical, customer facing, non-promotional roles within many pharmaceutical and biotechnology organizations [1]. MSL responsibilities have been variably described but generally involve scientific exchange with external stakeholders such as health-care professionals (HCPs), relaying insights to internal stakeholders, and supporting the generation of new evidence. For more detail, see Table S1 for guiding principles related to the MSL role, as established by the Canadian MSL Network, an informal association of professionals (Table S2).

Within organizations, metrics are a way to measure activity and can be used for many purposes. In addition to measuring progress toward an objective, metrics can also be used to assess resourcing and training needs, inform strategy, evaluate performance, assess impact, and communicate the value of a function within an organization. Metrics are commonly divided into two types: quantitative or qualitative, with the former characterizing quantity and the latter characterizing quality [2]. Both metric types have advantages and disadvantages. A quantitative metric, such as the number of continuing medical education programs supported, is relatively objective and easy to measure by counting. A qualitative metric, such as the quality of information contained in a continuing medical education program, in contrast, is more subjective and complex to measure. Both can fulfill important needs within an organization.

Despite its longstanding history [1], the complexity of the MSL role has made establishing appropriate metrics a challenge [3]. This is in contrast to the sales representative role, for which simple and firmly entrenched metrics exist. These metrics are typically quantitative and related to sales revenue and ‘reach and frequency’. However, common sales metrics are generally not applied to the MSL role as they may encourage inappropriate proactive outreach (i.e., approaching promotional) and/or oppose regulatory (Health Canada) [4] and industry (Innovative Medicines Canada) [5] guidance.

Internally, metrics relevant to field medical roles may vary by product lifecycle, therapeutic area, or strategic priorities of the organization, and are likely to have a longer time to vest [6]. External influences include increasingly complex health-care and payer environments, geographical spread (a particular challenge in Canada), and situational pressures within the health-care system (such as COVID-19). Apart from, but influenced by all of these factors, is the evolution of the role itself over time.

There is growing desire to use qualitative or outcomes-based assessments to address these complexities. Still, quantitative metrics, while not optimal determinants of impact, are generally simpler to measure than qualitative aspects of a role. For example, it is comparatively easier to record the ‘number of interactions’ an MSL has with an HCP than to assess and capture the impact of those interactions. For this reason, quantitative metrics may be used more often than, and sometimes serve as proxies for, qualitative outcomes.

In considering all of these points, it can be expected that, even within an organization or team, the metrics or key performance indicators (KPIs) applicable to MSLs will be numerous and heterogeneous. This makes it especially challenging for MSL leaders to succinctly communicate the value that their MSLs deliver to the organization.

Although surveys of MSL metrics have been conducted [7, 8], there has been no comprehensive assessment done within Canada. The Canadian environment is unique, with a blend of influences found in Europe (e.g., publicly funded health-care and health technology assessment) and in the USA (e.g., private health-care). Moreover, pricing reform [9], reimbursement challenges, and Health Canada’s increased scrutiny on industry practices [10], can influence how and why metrics are used.

As a first step toward guiding the strategic use of metrics for the MSL role, we—a sub-team of the Canadian MSL Network—conducted the following study, aiming to understand which, how, and why metrics are currently applied to MSLs working in Canada.

## Methods

To meet the objectives of this study, a web-based survey was created and disseminated by email invitation to pre-identified Canadian MSL leaders.

### Survey Population Identification

Target survey participants were MSL leaders working in Canada. To begin, the authors considered the definition of the MSL role established by the Canadian MSL Network (Table 1, Table S1). Next, MSL leaders were defined by the study team as individuals leading, overseeing, and/or managing MSLs (or equivalent titles), directly or indirectly, within the pharmaceutical and biotechnology space in Canada. This profile aligned well with the existing Canadian MSL Network membership, which served as the primary source of contact information (not publicly available) for participant invitations. To expand on this, MSL Network members were asked to refer their colleagues in MSL leadership roles. Finally, LinkedIn (Sunnyvale, CA, USA) was searched using the terms: “Canada” or “Ontario” or “Quebec”; and “medical” or “scientific”; and “leader”, or “director”, or “lead”, or “manager”. Unique results (i.e., MSL leaders who were not members of the Canadian MSL Network), were contacted about their interest in participating in the survey, but only if there was an existing professional connection between them and an author. Identification of and communication with potential participants met Personal Information Protection and Electronic Documents Act (PIPEDA) and Canadian Anti-Spam Legislation (CASL) regulations, since all contacts were obtained from the existing Canadian MSL Network membership or through professional connections.

**Table 1:** Definition of the Medical Science Liaison (MSL) role as defined by the Canadian MSL Network

1.	Industry professional
2.	Non-promotional role (i.e., reports to a Medical Affairs function)
3.	Predominantly field-based, customer facing
4.	Responsibilities include scientific exchange and Key Opinion Leader (KOL) interaction

Eligibility screening was conducted at the start of the survey (Questions 1, 2, and 3; see Table 2) and excluded participants who did not work in Canada, reported into a commercial (i.e., sales/marketing) function, or did not have leadership over MSLs. Individuals identified as ineligible within the survey were invited to refer colleagues who may be eligible. In total, 70 MSL leaders from 29 different pharmaceutical and biotechnology companies were identified for invitation to participate.

**Table 2:** MSL leader survey questions

Question
Informed consent
1. Do you work in Canada?^a^ (Yes/No = ineligible/terminate)
2. Please indicate which function you report into:^a^ (List includes other; Sales/Marketing = ineligible/terminate)
3. Which title best describes your current responsibilities?^a^ (List; I do not have leadership over MSLs = ineligible/terminate)
4. How many total years have you been in an MSL leadership role?^a^ (Number select)
5. How many MSLs report to you? Directly? Indirectly? (Number select)
6. What type of product do you/your MSL team support?^a^ (Select from list, all that apply)
7. At which life cycle stage(s) is/are the product(s) that your MSL team supports?^a^ (Select from list, all that apply)
8. Please rank the following MSL responsibilities according to their contribution to your organization:^a^ (Most to least important) a. Scientific engagement with HCPs b. Evidence generation c. Insight gathering
9. Please rank your agreement with the following statement: “Metrics are critical to understanding whether an MSL is delivering value”^a^ (Likert agreement scale)
10. Do you apply metrics to the MSL role?^a^ (Yes/No = skip to question 15)
11. Consider the reasons you apply metrics to the MSL role. Which are most important? Which are least important?^a^ (Assign importance to each reason listed)
12. a. Consider the following **quantitative metrics**. Over the past year, **which have you used** with your MSL(s)? (Select from list, all that apply)^a^ b. Considering the **quantitative metrics** that you collect, please indicate for which **reason(s) you collect them**. (Select from categories, all that apply)^a,b^ c. Considering the **quantitative metrics** that you collect, please indicate **who you share these metrics with**. (Select from list, all that apply)^a,b^ d. Consider each of these **quantitative metrics** again. In your opinion, how well does each **demonstrate the value of the MSL role**? (Very poorly to very well)^a,b^
13. a. Consider the following **qualitative metrics**. Over the past year, **which have you used** with your MSL(s)? (Select from list, all that apply)^a^ b. Considering the **qualitative metrics** that you collect, please indicate for which **reason(s) you collect them**. (Select from categories, all that apply)^a,c^ c. Considering the **qualitative metrics** that you collect, please indicate **who you share these metrics with**. (Select from list, all that apply)^a,c^ d. Consider each of these **qualitative metrics** again. In your opinion, how well does each **demonstrate the value of the MSL role**? (Very poorly to very well)^a,c^
14. Are there any other metrics (quantitative or qualitative) that you apply that were not mentioned previously? Please share. (Open text)
15. Considering the COVID-19 pandemic, do you expect metrics for MSLs to change in the future?^a^ (Yes/No)
16. Do you evaluate the quality of MSL contribution beyond metrics?^a^ (Yes/No)
17. Do you have any other comments or ideas related to how MSL leaders can communicate the value of the MSL role? (Open text)

See Table S4 for full questions and selections

*HCPs* health-care professionals, *MSL* Medical Science Liaison

^a^Indicates questions that are required to be answered (if applicable) to consider a response complete

^b^Responses from question 12a are piped into this question; i.e., metrics displayed are only those selected from 12a

^c^Responses from question 13a are piped into this question; i.e., metrics displayed are only those selected from 13a

### Survey Design

The online survey was designed by the authors, in line with expert guidance [11, 12], and built using the QuestionPro Inc. (Austin, TX, USA) survey software. Survey validation included face validity review by all authors and one QuestionPro researcher. In addition, the survey was piloted among the authors eligible to participate (i.e., current MSL leaders) to ensure technical and logical integrity as well as sound data collection and analytic capability. Results are reported according to the CHERRIES methodology (Table S3) [13].

The survey was anticipated to require approximately 30 minutes to complete. It was launched June 16th 2020, closed July 7th 2020, and was reopened (unadvertised) by request until July 15th, 2020. Invitations and reminders containing unique survey links, to prevent duplicate entry, were emailed via Campaign Monitor (Nashville, TN, USA) (Table S3). The survey and communications were provided in both official Canadian languages (English and French).

The survey contained 25 questions (Table 2 and Table S4) related to eligibility, demographics, and MSL metrics, with open-text options to collect written input where valuable. The questions in the survey referred to relevant activities of MSLs and were designed to assess the use and value of both quantitative and qualitative metrics applied to the MSL role. Value and impact were subjective, according to the participant’s interpretation. The majority of metrics listed were generated in a 2018 Canadian MSL Network metrics workshop, with additions made by the authors based on their recent professional experience. Since these lists were not considered exhaustive, the survey provided an open-text option where participants could provide additional metrics used within their organization. Questions were designed by the study authors using psychometrically appropriate question types [12, 14] from the QuestionPro software, including dichotomous, Likert scale, multiple choice, and matrix style questions. Selections were varied with drop-down, drag and drop, card sorting, slider, single-select and multiselect options offered. Logical options were randomized where possible (i.e., questions 8 and 11) to prevent question order effects. To reduce the number and complexity of the questions, adaptive questioning was applied (Table S3).

Survey participants were asked to reflect on metrics used over the past year, but before the COVID-19 pandemic. This timeframe was selected as the optimal recall window since it would reflect metrics used during a full fiscal cycle, minimize recall bias [15], and exclude variability caused by new ways of working resulting from the COVID-19 pandemic. Although no compensation was provided for participation, respondents were offered a preview of selected results prior to publication to encourage engagement. Completion was encouraged by applying validation to 19 out 24 questions (Table S3).

### Ethics

Ethics approval was not sought for this study as it was designed as a quality improvement initiative with no intervention [16]. An online consent form (Table S5) gated entry into the survey. The consent form provided information such as the study objectives, confidentiality, and data security precautions, and discouraged the sharing of personal or identifying information in open-text fields within the survey. Informed consent was mandatory in order to enter and participate in the survey.

### Data Collection and Storage

QuestionPro Inc. is General Data Protection Regulation (GDPR) [17] compliant and an ISO 27001:2013 [18] certified company. Data collected from the survey was stored on QuestionPro servers until November 2020, after which aggregate data was stored by the researchers according to their company policies.

Before launch, QuestionPro’s Respondent Anonymity Assurance feature was enabled to assign a computer-generated identification number to individual responses. This hid the following identifying information from researchers: respondent email, IP address, country code and region so that these attributes were never linked to response data. Company information was coded by an author and no responses were attributed to a specific individual nor company.

### Data Analysis

All results reported were compiled using the QuestionPro analytics dashboard or Microsoft Excel (Redmond, WA, USA) and were descriptive in nature (see Table S4 for the statistical application by question). Ineligible responses (i.e., answers to questions 1, 2 or 3 that resulted in termination of the survey, see Table 2) were not included in the study results. Responses from incomplete surveys were included in the overall analysis. Data from open-text fields were reviewed by the authors and rendered into general sentiments (rather than verbatim text) or summary lists (e.g., unique metrics mentioned and not previously listed). The response, participation, and completion rate calculations are explained in Table S3. Initial analyses included the use of pivot tables to split the cohort by years of experience (≤ 2 and > 2) and market type (specialty vs. mass) to assess variability within those segments. For analysis, metrics were grouped into categories related to the MSL role, namely; scientific engagement, insight gathering, evidence generation, or internal/operational (see Tables S7 and S8). Frequency of use for each metric was expressed as the proportion of respondents for that question selecting that metric. Responses to questions 12b and 13b (the reasons for applying metrics) and 12c and 13c (with whom they were shared) were expressed as the number of mentions within each subcategory (e.g., the number of times ‘to inform resourcing’ was selected) divided by the total number of mentions (e.g., total number of times any reason was selected). Metrics were considered to be of high value if the mean Likert scale result was ≥ 4.0 (i.e., demonstrate well or very well).

## Results

**Fig. 1:**
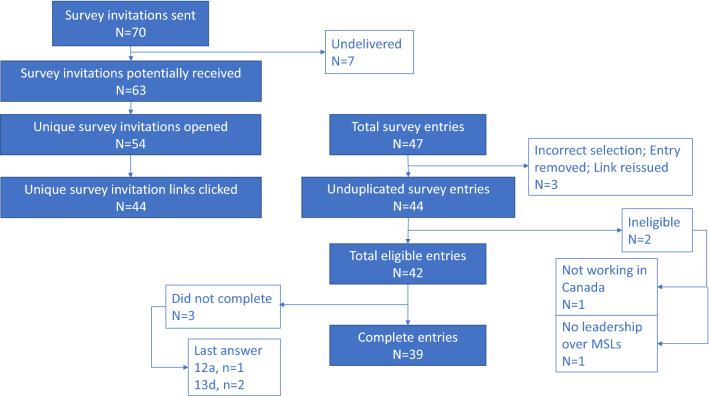
CONSORT diagram showing survey invitation, response and eligibility (Question 1, 2, 3)

Invitations were sent to 70 Medical Science Liaison (MSL) leaders from 29 Canadian companies; 36 contacts from 22 companies were sourced from the Canadian MSL Network list, and 34 contacts were sourced from referrals, including 7 additional companies. A further 14 companies were identified via LinkedIn as possibly having a medical liaison role, but no professional connections existed to enable outreach. Survey responses were received from 44 leaders (63%) and 22 companies (76%). Two respondents were ineligible, so the total number of participants was 42. Of these, 39 (93%) completed all questions (Fig. 1). The average time spent taking the survey was 28 min.

### Demographics

Table 3 details the demographics of the 42 leaders who participated in the survey. Nearly 86% had MSLs as direct reports, and two-thirds (*n* = 28/42, 67%) had ≤ 5 years’ total experience managing MSLs, with 45% having ≤ 2 years’ experience. Thirty-six leaders (86%) reported having teams that support specialty care products, while six (14%) had MSLs supporting only mass market products. With respect to product life cycle stage, the majority of respondents’ teams (86%) supported products from phase III until loss of exclusivity (LOE).

**Table 3:** Demographics (*N* = 42)

Variable	*N*	%
Years in MSL leadership role (Question 4)		
1–2	19	45.2
3–5	9	21.4
6–8	8	19.0
9–11	2	4.8
12–14	1	2.4
15+	3	7.1
MSL reporting type (Question 3)		
Direct reports	36	85.7
Indirect reports	6	14.3
Direct and indirect reports	2	4.8
Number of MSL reports (mean) (Question 5)		
Direct	5.6	
Indirect	7.7	
Market type(s) supported by MSL(s) (Question 6)		
Specialty care only	27	64.3
Specialty care and mass market/primary care	9	21.4
Mass market/primary care only	6	14.3
Product life cycle stage(s) supported by MSL(s) (Question 7)		
Phase I/II (i.e., early pipeline)	19	45.2
only	0	0.0
Phase III to pre-NOC (i.e., pre-launch)	33	78.6
only	0	0.0
Peri-launch (i.e., up to 2 years post-launch)	34	81.0
Only	0	0.0
2 years post-market up to LOE	34	81.0
Only	5	11.9
Post-LOE	5	11.9
Only	1	2.4

*LOE* loss of exclusivity

When comparing responses between those supporting mass market (*n* = 15) versus specialty care (*n* = 36), the only appreciable difference (data not shown) was a tendency for those supporting mass market to more frequently strongly agree that metrics are critical to understanding whether an MSL is delivering value (*n* = 4/15, 27% vs. *n* = 7/36, 19%). Likewise, those with > 2 years of experience (*n* = 23) were more likely to strongly agree with that statement (*n* = 6/23, 26%) than leaders with 1–2 years of experience (*n* = 2/19, 11%). Variability based on years of experience (1–2 years versus > 2 years) was also seen in the ranked importance of metrics for measuring progress on current medical tactics (*n* = 14/18, 78% versus *n* = 8/23, 35% ranked it high) and measuring individual MSL performance (*n* = 1/18, 6% versus 6/23, 26% ranked it low).

### Determining MSL Value via Metrics

**Fig. 2:**
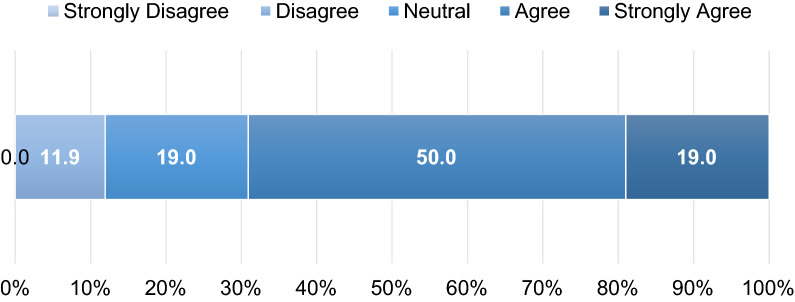
MSL leaders’ agreement with the statement: “metrics are critical to understanding whether an MSL is delivering value”, using a 5-point Likert scale (1 = strongly disagree, 5 = strongly agree) (*n* = 42) (Question 9)

**Fig. 3:**
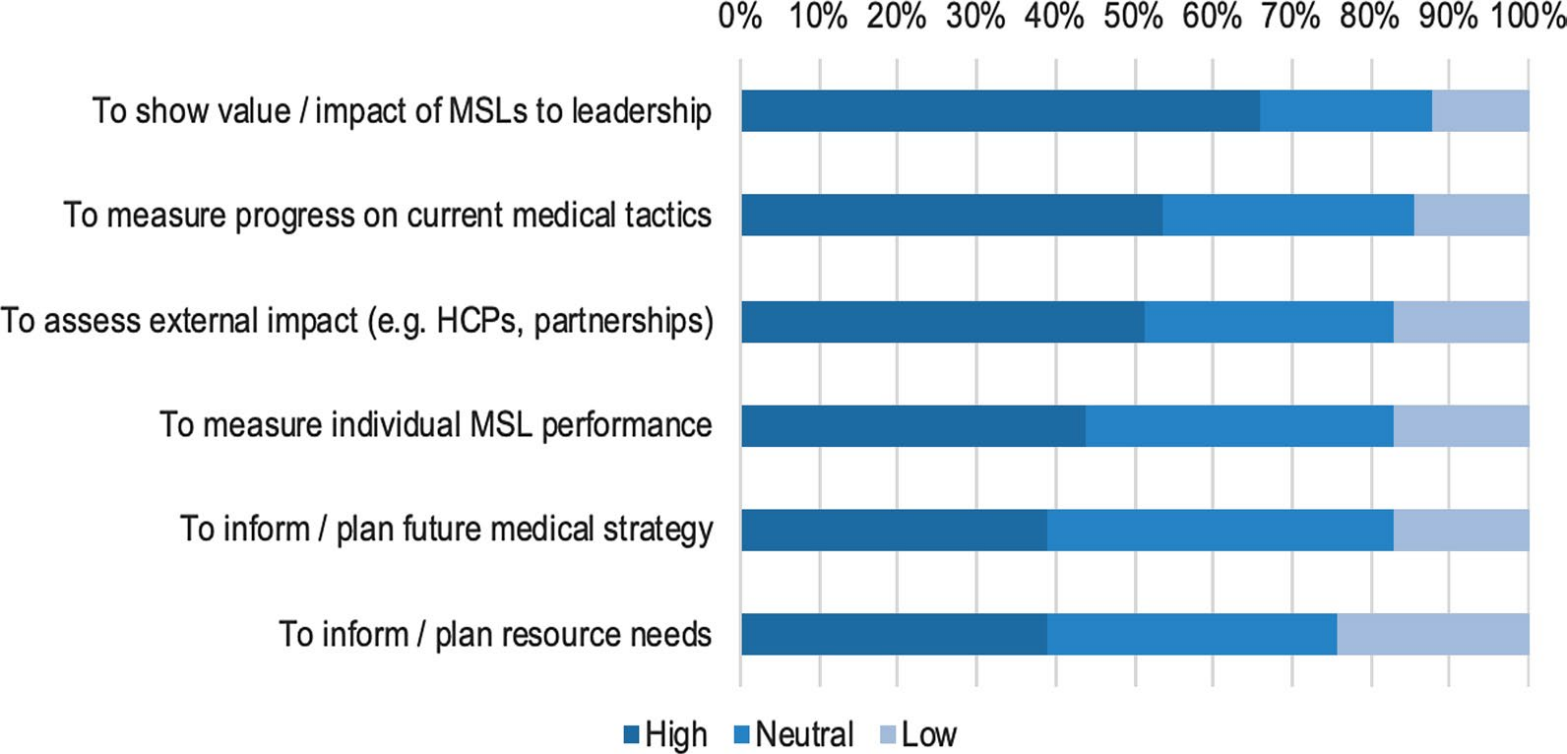
Reported importance of reasons for applying metrics to the MSL role (*n* = 41) (Question 11)

When asked to rank the importance of three MSL responsibilities (evidence generation, insight gathering, and scientific engagement) with respect to their contribution to their organization, 83% (*n* = 35/42) of respondents agreed that scientific engagement was the most important, followed by insight gathering (ranked second by 76%, *n* = 32/42) and evidence generation (ranked third by 93%, *n* = 39/42). Out of the MSL leaders surveyed (*n* = 42), 69% (*n* = 29/42) of leaders agreed or strongly agreed that metrics are critical to understanding whether an MSL is delivering value (Fig. 2). However, another survey question revealed that 88% (*n* = 37/42) of MSL leaders were using metrics, 10% (*n* = 4/42) were not using metrics at the time of the survey but had in the past year, and 2% (*n* = 1/42) had never utilized metrics. When respondents were asked to consider the reasons why they applied metrics to the MSL role, ‘to show value/impact of MSLs to leadership’ was most frequently ranked (66%, *n* = 27/41) as highly important and ‘to inform/plan resource needs’ was ranked least important most often (24%, *n* = 10/41) (Fig. 3, Table S6).

### Quantitative MSL Metrics

When grouped by metric type, 50% (*n* = 199) of quantitative metrics used (*n* = 395) were related to scientific engagement, 23% (*n* = 90) to internal/operational activities, 19% (*n* = 74) to insight gathering, and 8% (*n* = 32) to evidence generation. Weighted mean Likert rankings by category are shown in Table 4. Respondents were asked to identify which quantitative metrics they applied to their MSL teams, and how well each demonstrated the value of the MSL role (Table 4). The top collected scientific engagement-related metrics were ‘number of HCP interactions’, ‘number of interactions per HCP’ and the ‘number of HCPs per MSL (i.e., list size)’. The most highly valued metrics in this category were ‘length of HCP interactions’ (tracked by 49%), ‘number of speaker trainings supported’ (tracked by 44%) and the ‘number of partnerships established with HCPs’ (used by 22%). For internal/operational metrics, ‘number of internal activities’ was the most frequently collected; however, the most valued metric in the list was the ‘number of HCP plans generated’ (utilized by 17%). For the metrics in the insight gathering category, the ‘number of insights gathered’ by MSLs and the ‘number of advisory/consultancy meetings supported’ were collected by 68% and 61%, respectively. All four metrics in the insight gathering category were highly valued by the respondents. The most commonly tracked metric in the evidence generation category was the number of clinical trial site visits (tracked by 27%), and all metrics in that category, except the number of site visits, were highly valued.

**Table 4:** Categorization, frequency of use (survey question 12a, *N* = 41), and mean Likert ranking (survey question 12d, *N* = variable) for quantitative metrics

Metric category Metric	Number of selections (12a) (*N* = 41)	Percent of respondents (12a)	Number of respondents (12d)^a^	Mean Likert score (12d)
Scientific engagement				
Number of HCP interactions	38	92.7	37	3.4
Number of interactions per HCP	24	58.5	23	3.3
Number of HCPs per MSL (i.e., list size)	24	58.5	23	2.9
Length of customer interactions	20	48.8	20	4.1
Number of group HCP presentations	20	48.8	19	3.8
Number of speakers’ trainings supported	18	43.9	17	4.1
Number of CHE/OLA supported	17	41.5	16	3.6
Number of topics per interaction	13	31.7	12	3.4
Number of non-HCP interactions	11	26.8	11	3.1
Number of partnerships established with HCPs	9	22.0	9	4.0
Number of new HCPs seen	5	12.2	5	3.8
Sub category weighted mean Likert score				3.5
Internal/operational				
Number of internal activities (e.g., training support, presentations, material review, conference reports)	23	56.1	22	3.4
Time spent on internal activities	16	39.0	15	2.9
Budget—actual vs. target spend	15	36.6	14	2.9
Number of training/development activities (e.g., journal articles read, certifications completed)	11	26.8	10	2.9
Number of project milestones achieved	11	26.8	10	3.7
Number of HCP plans generated	7	17.1	7	4.1
Number of conferences attended	7	17.1	7	3.3
Sub category weighted mean Likert score				3.3
Insight gathering				
Number of insights gathered	28	68.3	27	4.0
Number of advisory/consultancy meetings supported	25	61.0	24	4.0
Number of insights actioned	13	31.7	13	4.7
Number of innovative ideas brought forward	8	19.5	7	4.7
Sub category weighted mean Likert score				4.2
Evidence generation				
Number of site visits	11	26.8	10	2.8
Number of research projects brought in for consideration	10	24.4	10	4.5
Number of research projects approved	5	12.2	5	4.0
Number of research projects managed	3	7.3	3	4.3
Number of new investigators/sites identified	3	7.3	2	4.0
Sub category weighted mean Likert score				3.8
Total (Likert score is weighted mean)	395		378	3.6

Options for 12d were piped from 12a; i.e., only metrics that were selected in 12a were asked about in 12d

^a^One respondent did not complete the survey past 12a, so some metrics are missing a response for 12d

### Qualitative MSL Metrics

Similar to quantitative metrics, the frequency of use and Likert agreement with usefulness in demonstrating the value of the MSL role are summarized for qualitative metrics in Table 5. Approximately 47% (*n* = 97) of qualitative metrics applied (*n* = 205) were associated with scientific engagement, 39% (*n* = 80) with internal/operational activities, and 14% (*n* = 28) with insight gathering. Weighted mean Likert rankings by category are shown in Table 5. There were no qualitative metrics associated with evidence generation as a selectable option. The most commonly used qualitative metric in the scientific engagement category was ‘HCP feedback’. It was also highly valued among respondents as a means of demonstrating the value of the MSL role. Other highly valued metrics in that category were ‘advocacy growth or stage of HCP relationship’ and ‘impact on patients’. The only metric in the insight gathering category, ‘quality of insights (as assessed by MSL manager)’, was tracked by 70% of respondents and was highly valued. Qualitative metrics associated with internal/operational activities included ‘cross-functional colleague feedback’, ‘medical colleague feedback’ and ‘qualitative description of impact’, all of which were deemed to demonstrate the value of the MSL role well or very well.

**Table 5:** Categorization, frequency of use (survey question 13a, *N* = 40), and mean Likert ranking (survey question 13d, *N* = variable) for qualitative metrics

Metric category Metric	Number of selections (13a) (*N* = 40)	Percent of respondents (13a)	Number of respondents (13d)	Mean Likert score (13d)
Scientific engagement				
HCP feedback (anecdotal, emails, etc.)	29	72.5	29	4.3
HCP assessment of value (from market research/survey)	23	57.5	23	4.0
Type of communication (in person, phone call, email, virtual)	23	57.5	23	3.4
Advocacy growth or stage of HCP relationship	12	30.0	12	4.6
Impact on patient (e.g., delivery of care, access to medicine, removing any barriers, educational needs supported) assessed by MSL manager	10	25.0	10	4.6
Sub category weighted mean Likert score				4.1
Internal/operational				
Cross-functional colleague feedback	37	92.5	37	4.0
Medical colleague feedback	31	77.5	31	4.1
Qualitative description of impact (e.g., STAR format, narrative)	12	30.0	12	4.3
Sub category weighted mean Likert score				4.1
Insight gathering				
Quality of insights (assessed by MSL manager)	28	70.0	28	4.4
Sub category weighted mean Likert score				4.4
Evidence generation				
Total (Likert score is weighted mean)	205		205	4.1

Options for 13d were piped from 13a; i.e., only metrics that were selected in 13a were asked about in 13d

### Using and Sharing Metrics

For metrics selected, respondents were asked their reasons for using each metric. The top reason for using quantitative metrics was to show the value/impact of MSLs to leadership, the top reason for using qualitative metrics was to measure individual MSL performance (data not shown). When asked with which functions quantitative and qualitative MSL metric reports were shared, senior medical leadership was the top recipient for both metric types (82%, *n* = 322/395 of quantitative metrics collected and 84%, *n* = 172/205 of qualitative metrics were shared with senior medical leadership). Approximately 55% of metrics (*n* = 216/395 quantitative and *n* = 112/205 qualitative) were shared with marketing, and 29% were shared with sales (*n* = 114/395 quantitative and *n* = 59/205 qualitative). ‘Number of HCP interactions’ was the most frequently used metric to show the value of the MSL to leadership (71%, *n* = 27/38) as well as the most frequently shared with senior medical leadership (84%, *n* = 32/38).

### Beyond Metrics and the Impact of the COVID-19 Pandemic

Respondents were asked if they evaluate the quality of MSL contributions beyond metrics, to which 79% (*n* = 31/39) indicated ‘yes’. A free text option was provided for respondents to elaborate, in which several themes emerged; an emphasis on the importance of collaboration with medical and cross-functional colleagues, the ability of the MSL to implement customer plans, and observation of MSLs during field visits with HCP customers. Some respondents (*n* = 6) noted the importance of sharing MSL achievements and impact with commercial and cross-functional units to not only demonstrate the value of the MSL, but to also drive support for medical resources.

Respondents were asked if they expect MSL metrics to change in the future given the COVID-19 pandemic and 74% (*n* = 29/39) expected they would. When asked to expand on their response in the free text space, a plurality of MSL leaders expected a permanent shift toward more virtual HCP interactions and to place more value on virtual engagements. Respondents also anticipated reductions in the number, frequency, and duration of HCP interactions, and thus greater emphasis on the quality of interactions. Moreover, with the shift to more virtual interactions, some respondents foresee adaptations to the MSL role including increasing territory/list sizes, or modifications to duties (e.g., more internal activities).

## Discussion

To our knowledge, this study is the first of its kind focused on the Canadian market. Drawing 44 respondents from 22 organizations, our survey results provide a unique perspective from MSL leaders within the Canadian pharmaceutical and biotechnology industry.

### Limitations

One common limitation of survey-based studies is sampling error [12]. With no reliable source of the number of pharmaceutical and biotechnology companies in Canada that employ MSLs, we were unable to estimate the total potential sample or characteristics of missing invitees. We therefore chose to use a variety of non-random sampling techniques (purposeful, convenience, and snow-balling) [12] and every effort was made (within legislation) to reach as many eligible participants as possible. Aided by the engaged membership of the longstanding Canadian MSL Network, our study boasted a high response rate (63%) from its distribution. Still, it is a limitation of our study that we cannot assess the degree of generalizability of our results to the broader MSL leader population.

In order to understand other potential sources of bias within the pool of survey respondents, results were observed between variable demographic cohorts. One of these was the predominance of MSL support for specialty markets (85%). Although analyses were not weighted according to any variable, results from a post hoc analysis of the data by market type (specialty versus mass) showed no substantially divergent answers for any single question except for the degree of strong agreement that metrics are critical. Likewise, the high proportion of respondents with less years of experience (45% had 1–2 years) responded similar to those with more experience for all but two questions. Finally, the majority of respondents (85%) identified that their MSLs support various phases of the product life cycle. Given that the observations within these cohorts were fairly consistent, the points presented herein are deemed to be relevant to our study population, irrespective of years of MSL leadership experience, market type, or product life cycle stage supported.

Another inherent boundary of our research is that it reflects only the perception of the MSL leader (which may vary depending on level within the organization) and the values of the MSL, higher leadership, or cross-functional colleagues were not assessed. Furthermore, technology to support the collection of metrics was not evaluated and may impact the ease or frequency of metric collection.

### Determining MSL Value via Metrics

To assess the perception related to the value of the MSL role, we sought to understand if there was consensus among leaders on the key contributions MSLs make to an organization. An important finding of this survey was that there was broad agreement that the primary responsibilities of MSLs were, in order, scientific engagement with HCPs, insight gathering, and evidence generation. Considering this, differences in metrics used and valued are unlikely to be due to different perceptions of the role and the value it delivers.

Metrics figure prominently in this Canadian MSL landscape, with 98% of respondents collecting or having collected metrics related to the MSL role over the past year. Interestingly, despite a high utilization of metrics, only 12% strongly agree that they are critical to understanding MSL value. This is the first datapoint that suggests a gap between metric collection and perceived value.

Our results confirm that metrics are collected for a variety of reasons, with each reason being deemed fairly important. However, the most frequently ranked reason, with the majority agreeing it was of high importance, was to show the value/impact of MSLs to leadership. Although the vast majority of respondents rated scientific engagement, an externally focused activity, as the most important MSL responsibility, only 51% of respondents placed a high importance on collecting metrics ‘to assess external impact’. This likely reflects the challenge related to assessing and capturing qualitative outcomes (i.e., impact), with many leaders emphasizing that while difficult to measure, such qualitative assessments are equally as important as quantitative measures.

### Metrics Collection and Sharing

The variety and variability of metrics collected was expected, given the complexity of the role and environment. Though companies are increasingly trying to incorporate qualitative and outcomes-based measures in their assessments [5–7], quantitative measures are still heavily relied upon. Some highly valued metrics such as the number of speaker trainings supported, partnerships established, impact on advocacy or patients, and those related to evidence generation may be less frequently collected due to differences in organizational structure (i.e., other functions within the organization support these activities). Others, particularly qualitative metrics, may be less commonly collected simply because of the difficulty associated with collecting them. It is important that these metrics not be overlooked, but considered by leaders in the context of their organizations.

Number of HCP interactions was the single-most collected metric surveyed (93%) and was associated with a moderate value in terms of demonstrating the value of the role (mean Likert score 3.4). Yet, it was the metric most frequently shared with medical leadership and the most frequently used with the intent of showing value. This example underscores a trend observed throughout the results, with misalignment between frequently collected metrics and those that were perceived as being the best demonstrators of value of the MSL role.

An alternate metric that may deserve further application for assessing scientific engagement is ‘length of customer interaction’. This measure was felt to show the contribution of the MSL well. It is a quantitative metric that could act as a proxy for quality, considering longer interactions may reflect value derived by an HCP and/or more opportunity for insight exchange. In terms of qualitative metrics, HCP feedback was both commonly collected and highly valued and therefore worthy of continued use. Still, there remains a gap concerning metrics that adequately reflect the value delivered by scientific engagement and its impact on the external environment.

Interestingly, while not all frequently collected, all of the metrics related to insight gathering were seen as excellent for demonstrating the value of the MSL. This is perhaps because insight gathering reflects both a quality of relationship with external stakeholders and tangible value to internal stakeholders. We would therefore encourage leaders to further develop, use and share these insight-related metrics.

The high utilization of internal feedback from medical and cross-functional colleagues reflects open-text comments that underlined the importance of collaboration in field-based medical roles. However, opportunity exists to further refine how internal impact of MSLs is measured. Another highly valued internal/operational metric was ‘number of HCP plans generated’, although not commonly utilized (17%). Metrics related to the generation of such plans and their execution have great potential in strategically aligning internal and external objectives and MSL contributions.

It was encouraging to see that metrics collected are commonly shared, and most often with medical leadership. If more highly valued metrics are explored and implemented it can be expected that the strategic use and sharing of metrics might increase.

### Beyond Metrics and the Impact of the COVID-19 Pandemic

Ultimately, it is important to recognize that 80% of respondents indicated that there is value to the MSL role that cannot be captured by existing metrics. Interestingly, the suggestions for filling the gap were qualitative in nature. Perhaps some could be fulfilled using existing or novel qualitative measures. Nevertheless, metrics serve as only one of a variety of tools available to leaders to both understand and communicate the value that their teams deliver. This underscores the value and importance of MSL managers themselves; to use these tools to build effective teams that are valued within the organization.

It is clear that the environment is evolving and metrics will have to as well. Perhaps the most immediate change will be in response to the COVID-19 pandemic. Respondents indicated they anticipate a shift toward digital engagement and as such we can expect that metrics surrounding this medium would increase in prominence and value. The timing of these results intersects with an important time of adaptation while approaches to customer engagement are rapidly changing as a result of the COVID-19 pandemic. There is now a critical need to engage differently and leverage digital platforms to meet the scientific needs of the medical community. In Canada, this presents an opportunity to expand geographic footprints so that the needs of under-serviced communities can now be met.

## Conclusion

This work underscores the collective trust and transparency of MSL leader participants and represents the largest survey of its kind in Canada. From the results, we learned that scientific engagement is regarded as the highest value and impact that the MSL role brings. However, it was consistently seen that the metrics most commonly collected do not grasp the true essence of the role and that there is a strong desire, among MSL leaders who responded, for more meaningful measures of impact. The results also indicate that the predominance of metrics used reflect a traditional quantitative approach. However, it is clear that the respondents are challenging norms to find novel ways to capture and communicate true impact.

Collectively, this data provides a baseline against which progress can be measured going forward and it provides directional focus on where valuable improvement can be made related to metric collection and use to better measure the impact of MSLs. Further research is needed to continue to understand how the industry is evolving its approach to measuring the value the MSL provides. To this end, we recommend future studies assess the impact of digital tools, advanced analytics, or artificial intelligence on the use and value of metrics applied to the MSL role.

## Supplementary Information

Below is the link to the electronic supplementary material.

Supplementary file1 (DOCX 68 kb)
