# Supplementary material for: Evaluating Metrics Applied to the Medical Science Liaison (MSL) Role: A Survey-Based Study of Canadian MSL Leaders
Source: Ther Innov Regul Sci. 2021 May 4;55(5):954–65. doi: 10.1007/s43441-021-00291-y (PMC8095215; doi:10.1007/s43441-021-00291-y)
Supplement: Supplementary file 1 — Supplementary file1 (DOCX 68 kb) [file 43441_2021_291_MOESM1_ESM.docx]

# **Supplementary Information**

**Table S1** Guiding Principles of the MSL role, as described by the Canadian Medical Science Liaison Network

| 1. Is a non-promotional role, as dictated by legal statutes, regulatory guidelines, and external and internal policies. 2. Reports to a non-sales/marketing functional line (e.g., Medical). 3. Provides scientific exchange according to external stakeholder needs, through communication that is fair-balanced (risk-benefit), unbiased, and evidence-based. 4. Gathers and translates insights from external stakeholders into actionable feedback to inform medical and brand strategies. 5. Facilitates medical and scientific collaborations (including both research- and educational-based partnerships) between healthcare providers, researchers, and industry. 6. Develops mutual needs-based relationships with external stakeholders to help inform decisions and optimal access to approved medicines, with the goal of improving the quality of care for Canadian patients. |
| --- |

**Table S2** Description of the Canadian Medical Science Liaison Network

| **The Canadian Medical Science Liaison Network**  Initiated nearly a decade ago in 2011, this group was formed by individuals from research-based pharmaceutical and biotechnology companies across Canada. The objective of the group was and is to provide a forum for the open exchange of ideas and information of a non-confidential nature related to Field Medical between participating companies and promote a closer, professional, and non-competitive relationship amongst Field Medical Personnel. In addition the Forum continuously monitors the industry and external environment for trends in the area of Field Medical and acts as a reference to associates that interface with regulatory agencies or other governing bodies related to operating standards and guiding principles of Field Medical personnel. |
| --- |

# **Table S3** CHERRIES methodology (https://www.jmir.org/2004/3/e34/pdf)

| **Design** | Describe survey design | Target population: Canadian MSL leaders.  Sample: Members of the MSL Network and professional contacts, by invite only. |
| --- | --- | --- |
| **IRB (Institutional Review Board) approval and informed consent process** | IRB approval | As this was a quality improvement initiative with no intervention, IRB approval was not sought. |
|  | Informed consent | Consent was requested at the beginning of the survey (see Table S5 for a copy of the consent form). |
|  | Data protection | Names and email addresses were entered into the QuestionPro database in order to generate unique survey links and participant IDs. According to the QuestionPro Privacy (https://www.questionpro.com/help/1.html) and Security (https://www.questionpro.com/images/qphome/QuestionPro-Security-Policy-Procedures.pdf) policies, all data and e-mail addresses uploaded to the system are owned solely by the survey administrators and never shared with any 3rd parties. Physical security features include the use of QuestionPro owned and managed servers that are co-located in off-site data centers that undergo periodic SSAE 16 SOC audits and are monitored for unauthorized access and service availability twenty-four hours a day. All customer data, including the data of end-users and administrators, is logically separated by account-based rules that require the entry of a unique username and password with each login. |
| **Development and pre-testing** | Development and testing | The survey was designed by the research team, in consultation with experts at QuestionPro. A test was sent to all authors to test usability and technical functionality before wide dissemination. |
| **Recruitment process and description of the sample having access to the questionnaire** | Open survey versus closed survey | Ours was a closed survey, by invitation (unique link) only. |
|  | Contact mode | Email invitation (see Table S9). |
|  | Advertising the survey | Not advertised. |
| **Survey administration** | Web/E-mail | The survey was web-based. |
|  | Context | The survey was hosted on QuestionPro.com and accessed only for the purpose of taking the survey. The software used was the Research package and data was captured automatically in the QuestionPro database. |
|  | Mandatory/voluntary | Voluntary. |
|  | Incentives | No monetary incentives were offered for completion. Participants were offered a view of high level survey results following analysis. |
|  | Time/Date | The survey was open for 3 weeks (June 16th to July 7th 2020, and reopened (unadvertised) by request until July 15th 2020). |
|  | Randomization of items or questionnaires | Options for questions 8 & 11 were randomized using the Question Pro software to prevent biases. |
|  | Adaptive questioning | To reduce the number and complexity of the questions, the following were applied:  Consent: Answer “I decline to participate” = terminate  Q1. Answer “No” = terminate  Q2. Answer “Sales/Marketing” = terminate  Q3. Answer “I do not have leadership over MSLs” = terminate  Q10. Answer “No” = skip to Q15  Q12a. Only selections from “a” are piped as options for 12b., 12c., and 12d.  Q13a. Only selections from “a” are piped as options for 13b., 13c., and 13d. |
|  | Number of Items | Each question had its own dedicated screen / page or prior answers were hidden to highlight the active question in that section. |
|  | Number of screens (pages) | The survey was presented over 20 pages / screens. |
|  | Completeness check | Validation (a software feature where respondents are prompted to answer missing questions before moving on to the next) was turned on for the following questions: Consent, 1, 2, 3, 4, 6, 7, 8, 9, 10, 11, 12a, 12b, 12d, 13a, 13b, 13d, 15 and 16.  All validated questions offered a non-response option (“not applicable” or “none”) where logical. |
|  | Review step | Although there was no review offered at the end of the survey, there was the option to go back and modify responses. |
| **Response rates** | Unique site visitor | Response rate was determined by the number of unique respondents divided by the number of invitations sent. Unique visitors were determined by unique survey links for each invitee. |
|  | View rate (Ratio of unique survey visitors/unique site visitors) | Not applicable. |
|  | Participation rate (Ratio of unique visitors who agreed to participate/unique first survey page visitors) | The number of people agreed to participate (consented), divided by visitors who visit the first page of the survey. For our survey this was 44/44 = 100%. |
|  | Completion rate (Ratio of users who finished the survey/users who agreed to participate) | The number of people submitting the last questionnaire page, divided by the number of people who agreed to participate (or submitted the first survey page). For our survey this was 41/44 = 93.2%. |
| **Preventing multiple entries from the same individual** | Cookies used | No. |
|  | IP check | No. |
|  | Log file analysis | No. |
|  | Registration | Unique survey links for each user gated entry into the survey. |
| **Analysis** | Handling of incomplete questionnaires | Incomplete questionnaires were included in the analysis, with N numbers in the results indicating completeness of response. |
|  | Questionnaires submitted with an atypical timestamp | No responses were excluded based on atypical timestamp (i.e. there was no minimum time required to take the survey) |
|  | Statistical correction | No weighting of items or propensity scores were used to adjust for the non-representative sample. |

# **Table S4** Survey questions, Type and Selections, and Statistical Analysis applied

| Question | Type  Selections | Statistical |
| --- | --- | --- |
| Informed Consent  See Table S5 | Select One   - I agree to participate, take me to the survey! - I decline participation = terminate | None |
| 1. Do you work in Canada?* | Select One   - Yes - No = ineligible/terminate | Count |
| 1. Please indicate which function you report into:* | Select One   - Medical / Scientific Affairs - Sales / Marketing = ineligible/terminate - Other (free text) | Count |
| 1. Which title best describes your current responsibilities?* | Select One   - Leader with MSLs as direct reports - Leader with MSLs as indirect reports (e.g. executive level) - Both of the above - I do not have leadership over MSLs = ineligible/terminate | Count |
| 1. How many total years have you been in an MSL leadership role?* | Drop-down Menu   - Nominal, 1-14 and 15+ | Count, Percent, Mean, SD, Variance  Post-hoc: grouped 1-2 and >2 years |
| 1. How many MSLs report to you? | Numeric Slider   - Directly (1 to 30) - Indirectly (1 to 30) | Count and Mean, SD, Variance for each |
| 1. What type of product do you / your MSL team support?* | Select Many   - Mass market / Primary care (e.g. diabetes, cardiovascular) - Specialty care (e.g. oncology, niche area of a mass market) | Count and Percent for each and overlap |
| 1. At which life cycle stage(s) is/are the product(s) that your MSL team supports?* (Select from list, all that apply) | Select Many   - Phase I/II (i.e. early pipeline) - Phase III to pre-NOC (i.e. pre-launch) - Peri-launch (i.e. up to 2 years post-launch) - 2 years post-market up to LOE (loss of exclusivity) - Post-LOE (loss of exclusivity) | Count and Percent for each and overlap |
| 1. Please rank the following MSL responsibilities according to their contribution to your organization:* (1=Most important; 3=least important)    - Scientific engagement with HCPs    - Evidence Generation    - Insight Gathering | Drag and Drop (options randomized)  1:  2:  3: | Count and Percent for each rank/responsibility combination |
| 1. Please rank your agreement with the following statement: “Metrics are critical to understanding whether an MSL is delivering value”* | Text Slider, Interval (Likert scale)  1- Strongly Disagree  2- Disagree  3- Neutral  4- Agree  5- Strongly Agree | Count and Percent for each  Mean, SD, Variance overall |
| 1. Do you apply metrics to the MSL role?* | Select One   - Yes - Not currently, but I have in the past year - No (skip to 15) | Count and Percent for each |
| 1. Consider the reasons you apply metrics to the MSL role. Which are most important? Which are least important?*  - To inform / plan future medical strategy - To measure progress on current medical tactics - To show value / impact of MSLs to leadership - To assess external impact (e.g. HCPs, partnerships) - To inform / plan resource needs - To measure individual MSL performance | Card Sorting Closed (options randomized)  Low importance:  Neutral importance:  High Importance: | Count and Percent for each reason/importance combination |
| 1. 1. Consider the following **quantitative metrics**. Over the past year, **which have you used**with your MSL(s)? (Select all that apply)* | Select Many  (see list from Table 4, plus 'none’) | Count and Percent for each  Total number of selections |
| - 1. Considering the **quantitative metrics**that you collect, please indicate for which **reason(s) you collect them**. (Select from categories, all that apply)*^1^ | Checkbox / Multi-select   - To inform / plan future medical strategy - To measure progress on current medical tactics - To show value / impact of MSLs to leadership - To assess external impact (e.g. on HCPs) - To inform / plan resourcing needs - To measure individual MSL performance - Other / N/A | Count and Percent for each  Count and Percent by category |
| - 1. Considering the **quantitative metrics**that you collect, please indicate **who you share these metrics with**. (Select all that apply)*^1^ | Checkbox / Multi-select   - Senior Medical Leadership - Marketing - Sales - Market Access / Government Relations - Clinical Affairs / Operations - Other | Count and Percent for each  Count and Percent by category |
| 12.   - 1. Consider each of these **quantitative metrics**again. In your opinion, how well does each **demonstrate the value of the MSL role**?*^1^ | Multipoint Scales, Interval (Likert Scale)  1- Very Poorly  2- Poorly  3- Neutral  4- Well  5- Very Well | Count and Percent for each  Mean, SD, Variance for each  Weighted mean by category and total |
| - 1. Consider the following **qualitative metrics**. Over the past year, **which have you used** with your MSL(s)? (Select all that apply)* | Select Many  (see list from Table 5, plus 'none’) | Count and Percent for each  Total number of selections |
| 13.   - 1. Considering the **qualitative metrics** that you collect, please indicate for which **reason(s) you collect them**. (Select from categories, all that apply)*^2^ | Checkbox / Multi-select   - To inform / plan future medical strategy - To measure progress on current medical tactics - To show value / impact of MSLs to leadership - To assess external impact (e.g. on HCPs) - To inform / plan resourcing needs - To measure individual MSL performance - Other / N/A | Count and Percent for each  Count and Percent by category |
| 13.   - 1. Considering the **qualitative metrics** that you collect, please indicate **who you share these metrics with**. (Select all that apply)*^2^ | Checkbox / Multi-select   - Senior Medical Leadership - Marketing - Sales - Market Access / Government Relations - Clinical Affairs / Operations - Other | Count and Percent for each  Count and Percent by category |
| - 1. Consider each of these **qualitative metrics** again. In your opinion, how well does each **demonstrate the value of the MSL role**?*^2^ | Multipoint Scales, Interval (Likert Scale)  1- Very Poorly  2- Poorly  3- Neutral  4- Well  5- Very Well | Count and Percent for each  Mean, SD, Variance for each  Weighted mean by category and total |
| 1. Are there any other metrics (quantitative or qualitative) that you apply that were not mentioned previously? Please share. | Comment Box  (no character limit) | No formal analysis |
| 1. Considering the COVID-19 pandemic, do you expect metrics for MSLs to change in the future?* | Select One   - Yes > Please elaborate (comment box) - No | Count and Percent for each  No formal analysis for comments |
| 1. Do you evaluate the quality of MSL contribution beyond metrics?* | Select One   - Yes > How? Please elaborate (comment box) - No | Count and Percent for each  No formal analysis for comments |
| 1. Do you have any other comments or ideas related to how MSL leaders can communicate the value of the MSL role? (Open text) | Comment Box  (no character limit) | No formal analysis for comments |

* indicates questions that are required to be answered to complete the survey

HCPs, healthcare professionals; NOC, Notice of Compliance (Health Canada); SD, standard deviation

# **Table S5** Online Survey Consent Form

| **Informed Consent to Participate in Research**  We are inviting you to take a survey for research. This survey is completely voluntary; there are no negative consequences if you decide not to participate. However, if you start the survey, the data related to your entry is anonymized and can therefore not be withdrawn. Please review all of the information below before deciding whether or not you would like to participate.  Study title:  Understanding current use and value of metrics related to the Medical Science Liaison (MSL) role; a survey of Canadian MSL leaders.   Researchers and funding:  The Canadian MSL Network members responsible for the cost, design, conduct, analysis and reporting of this survey include: Lisa Cesario (Hoffmann-La Roche Ltd), Andrew Chilelli (Astellas Pharma), Simon Collin (Abbvie), Marsha Haynes (Janssen), Peter Langlois (AstraZeneca), Munaza Saleem (Hoffmann-La Roche Ltd), and Lisa Wilcox (Pfizer). The project is also supported by an independent medical writer, Stevie Kenyon (Placencia Holdings Ltd).   Study objective:  To understand the current use and perceived value of metrics related to the MSL role, according to Canadian MSL leaders from various companies across the innovative pharmaceutical/biotechnology industry. The intent of the study is Quality Improvement.   Estimated number of participants: 50  What is my involvement?  In this survey you will be asked general questions about your professional role and your team. You will then be asked detailed questions regarding metrics applied to the MSL role, including your opinion of which are valuable or not. The survey will take approximately 30 minutes of your personal time.  Are there any risks related to my participation?  We do not anticipate any physical, psychological/emotional, financial, or social risks related to your participation.  Confidentiality and Data Security  While we cannot eliminate all risks related to sharing information online, we have done our best to plan for and minimize risk where we can by:   1. using a secure system to collect and store data (see: Question Pro [security](https://www.questionpro.com/security/)) until November 2020. Thereafter, aggregate data will be stored by the researchers according to their company policies. 2. enabling QuestionPro [Respondent Anonymity Assurance](https://www.questionpro.com/security/raa.html), which assigns a computer generated identification number to individual responses and hides the following from researchers: Respondent Email, IP Address, Country Code and Region. These attributes are therefore not linked to response data. Company information is also coded and no responses will be attributed to a specific company. To support these efforts, please do not share any personal or identifying information when answering in open text fields.   Note: Entry into the survey is considered consent to use the data you provide. Due to the confidential nature of the study, data cannot be removed once entered (i.e. partial or unsubmitted responses may be included in the analysis).  Who can see my data?   - Your contact information (name, employer, and company email address) was obtained through the MSL Network. - We (the researchers) will have access to coded company and de-identified (no names, emails, IP address, or region) data from the survey. This protects your privacy while enabling data analysis and study conduct. - We may share our findings in publications or presentations. If we do, the results will be aggregate (grouped) and/or de-identified data (no link to name, company, etc.). - De-identified data may be shared with other researchers in the future. - Open text responses will not be shared verbatim in the public domain, but sentiments and themes may be.   Possible benefits:  Reading the survey content may help you reflect on the value of current metrics used in evaluating MSL activities and may give you ideas to implement with your team going forward. The questions in the survey refer to relevant activities of field-based MSLs. We recognize it may not be all encompassing, however, it reflects the collective thinking from a variety of sources (Canadian and beyond) to help inform the focus for this survey. The metrics mentioned are not suggestions, guidelines, codes, rules or laws. Respondents should refer to their employer and industry guidelines for relevant codes of conduct, laws and regulations.  You will receive preliminary study results via your MSL Network membership and a link to the final publication for your reference.  Compensation: None.    Questions about the research, complaints, or problems?  Contact: Lisa Cesario  Medical Strategy Director, Medical and Regulatory Affairs  Hoffmann-La Roche Limited  7070 Mississauga Road  Mississauga, ON  L5N 5M8 Canada  Mobile: 647-289-4489  Mail to: lisa.cesario@roche.com  Eligibility  To take this survey, you must be working in Canada and leading / managing Medical Science Liaisons.  Agreement to participate  Your participation is completely voluntary.   - I agree to participate, take me to the survey! - I decline participation. |
| --- |

**Table S6** Ranked importance of various reasons MSL leaders use metrics for the MSL role

|  | To inform / plan future medical strategy | | To measure progress on current medical tactics | | To show value / impact of MSLs to leadership | | To assess external impact (e.g. HCPs, partnerships) | | To inform / plan resource needs | | To measure individual MSL performance | |
| --- | --- | --- | --- | --- | --- | --- | --- | --- | --- | --- | --- | --- |
|  | N | % | N | % | N | % | N | % | N | % | N | % |
| Low importance | 7 | 17.1% | 6 | 14.6% | 5 | 12.2% | 7 | 17.1% | 10 | 24.4% | 7 | 17.1% |
| Neutral importance | 18 | 43.9% | 13 | 31.7% | 9 | 22.0% | 13 | 31.7% | 15 | 36.6% | 16 | 39.0% |
| High importance | 16 | 39.0% | 22 | 53.7% | 27 | 65.9% | 21 | 51.2% | 16 | 39.02% | 18 | 43.9% |

**Table S7** Categorization for quantitative metrics (question 12a)

|  |
| --- |
| **Scientific engagement** |
| # of HCP interactions |
| # of interactions per HCP |
| # of HCPs per MSL (i.e. list size) |
| Length of customer interactions |
| # of group HCP presentations |
| # of speakers’ trainings supported |
| # of CHE/OLA supported |
| # of topics per interaction |
| # of non-HCP interactions |
| # of partnerships established with HCPs |
| # of new HCPs seen |
|  |
| **Internal / Operational** |
| # of internal activities (e.g. training support, presentations, material review, conference reports) |
| Time spent on internal activities |
| Budget – actual vs. target spend |
| # of training / development activities (e.g. journal articles read, certifications completed) |
| # of project milestones achieved |
| # of HCP plans generated |
| # of conferences attended |
|  |
| **Insight gathering** |
| # of insights gathered |
| # of advisory / consultancy meetings supported |
| # of insights actioned |
| # of innovative ideas brought forward |
|  |
| **Evidence generation** |
| # of site visits |
| # of research projects brought in for consideration |
| # of research projects approved |
| # of research projects managed |
| # of new investigators / sites identified |
|  |

**Table S8** Categorization for qualitative metrics (question 13a).

|  |
| --- |
| **Scientific engagement** |
| HCP feedback (anecdotal, emails, etc.) |
| HCP assessment of value (from market research/survey) |
| Type of communication (in person, phone call, email, virtual) |
| Advocacy growth or stage of HCP relationship |
| Impact on patient (e.g. delivery of care, access to medicine, removing any barriers, educational needs supported) assessed by MSL manager |
|  |
| **Internal / Operational** |
| Cross-functional colleague feedback |
| Medical colleague feedback |
| Qualitative description of impact (e.g., STAR format, narrative) |
|  |
| **Insight gathering** |
| Quality of insights (assessed by MSL manager) |
|  |
| **Evidence generation** |
|  |

**Table S9** Templates of invitations sent using Campaign Monitor (Nashville, TN, USA) to pre-identified Canadian MSL leaders.

| **SURVEY INVITATION EMAIL** |
| --- |
| Date: Jun 16, 2020  Sent from: lisa.cesario@roche.com  Subject Line: The Canadian MSL Network wants to hear from you! / Le Réseau Canadien de MSL souhaite connaître votre opinion!  Keywords: MSL metrics survey / Sondage d’évaluation des MSL  ----- Communication française à suivre ------  Dear <first name>    Thank you for being a part of the Canadian Medical Science Liaison (MSL) Network. Today, we are excited to invite you to participate in our first national, industry-wide survey of MSL leaders. Insights you share in this survey will illustrate what, how, any why metrics are used to measure MSL activities across the country. Join us, as we establish a broader appreciation for the MSL role and how to best measure its value.    The survey will take approximately 20 minutes to complete and is best viewed on a computer or tablet. The survey will be open until **July 7, 2020**. Your responses are anonymized. More information can be found on the first page of the survey prior to your consent.    “Take Survey Now” (image link)    **This link is unique to you, so please do not share it with others.** If you know of other MSL leaders who may not have received an invitation, please encourage them to contact us to request their own unique survey invitation link.  In appreciation of your participation, we will share select results with you in advance of publishing the full study results.    If you have any questions or concerns, please do not hesitate to contact us. Thank you, in advance, for your contribution.    Sincerely,    **Lisa Cesario** on behalf of **the Canadian MSL Network**  Medical Strategy Director  Medical and Regulatory Affairs  Hoffmann-La Roche Limited  7070 Mississauga Road  Mississauga, ON L5N 5M8 Canada  Mobile: 647-289-4489  Mail to: lisa.cesario@roche.com    For technical support, contact [ashok.singh@questionpro.com](mailto:ashok.singh@questionpro.com) at QuestionPro.    **----------**  Cher/Chère <first name>  Nous vous remercions de faire partie du Réseau Canadien d’agents de liaison Médicoscientifique (MSL). Nous sommes heureux aujourd’hui de vous inviter à participer à notre sondage à l’échelle nationale auprès des chefs des MSL. Les opinions exprimées dans ce sondage permettront d’illustrer les données utilisées ainsi que la manière dont elles sont employées et les raisons pour lesquelles elles sont employées pour évaluer les activités des MSL à l’échelle nationale. Joignez-vous à nous pour nous aider à mieux comprendre le rôle des MSL et la meilleure façon de mesurer leur valeur.  Le sondage prend environ 20 minutes à remplir et sera mieux consultée sur un ordinateur ou une tablette. Le sondage sera ouverte jusqu'au **7 juillet 2020**. Vos réponses sont anonymisées. De plus amples renseignements figurent sur la première page du sondage avant la section de consentement.   « Remplir le sondage maintenant » (lien visuel)  **Ce lien vous étant exclusivement réservé, veuillez donc ne pas le transmettre à autrui.** Si vous connaissez des chefs de MSL qui n’ont pas reçu d’invitation, veuillez les encourager à communiquer avec nous pour recevoir un lien unique d’invitation au sondage.  Pour vous remercier de votre participation, nous vous ferons part de certains résultats avant la publication de l’ensemble des résultats du sondage.  Si vous avez des questions ou des préoccupations, n’hésitez pas à communiquer avec nous. Nous vous remercions à l’avance de votre contribution.  Cordialement,  **Lisa Cesario** au nom du **Réseau Canadien de MSL**  Directrice, Stratégies médicales  Affaires médicales et réglementation  Hoffmann-La Roche Limitée  7070 Mississauga Road  Mississauga (Ontario)  L5N 5M8 Canada  Cellulaire : 647-289-4489  Courriel : lisa.cesario@roche.com  Pour obtenir du soutien technique, envoyez un courriel à QuestionPro à l’adresse suivante : [ashok.singh@questionpro.com](mailto:ashok.singh@questionpro.com).  © 2020 Hoffmann-La Roche Ltd. All Rights Reserved.  [Unsubscribe](https://rochecanada.cmail20.com/t/r-u-shhttj-oyuqlkdkd-c/) |
| **MID-REMINDER EMAIL (x2)** |
| Date: Jun 23, 2020 and June 30, 2020  Sent from: lisa.cesario@roche.com  Subject Line: The Canadian MSL Network (still) wants to hear from you! / Le Réseau Canadien de MSL souhaite (toujours) connaître votre opinion!  Keywords: MSL metrics survey / Sondage d’évaluation des MSL    ----- Communication française à suivre ------  Dear <first name>    This is a friendly reminder that the Canadian Medical Science Liaison (MSL) Network survey of MSL leaders is open and awaiting your participation.    If you have already taken the survey, please disregard this email.  The survey will take approximately 20 minutes to complete and is best viewed on a computer or tablet. The survey will be open until **July 7, 2020**. Your responses are anonymized. More information can be found on the first page of the survey prior to your consent.    “Take Survey Now” (image link)    **This link is unique to you, so please do not share it with others.** If you know of other MSL leaders who may not have received an invitation, please encourage them to contact us to request their own unique survey invitation link.  In appreciation of your participation, we will share select results with you in advance of publishing the full study results.    If you have any questions or concerns, please do not hesitate to contact us. Thank you, in advance, for your contribution.    Sincerely,    **Lisa Cesario** on behalf of **the Canadian MSL Network**  Medical Strategy Director  Medical and Regulatory Affairs  Hoffmann-La Roche Limited  7070 Mississauga Road  Mississauga, ON L5N 5M8 Canada  Mobile: 647-289-4489  Mail to: lisa.cesario@roche.com    For technical support, contact [ashok.singh@questionpro.com](mailto:ashok.singh@questionpro.com) at QuestionPro.  ----------  Cher/Chère <first name>  Nous souhaitons seulement vous rappeler que le sondage auprès des chefs des MSL du Réseau Canadien d’agents de liaison Médicoscientifique (MSL) se déroule actuellement et nous vous saurions gré de bien vouloir y participer.  Le sondage prend environ 20 minutes à remplir et sera mieux consultée sur un ordinateur ou une tablette. Le sondage sera ouverte jusqu'au **7 juillet 2020**. Vos réponses sont anonymisées. De plus amples renseignements figurent sur la première page du sondage avant la section de consentement.   « Remplir le sondage maintenant » (lien visuel)  **Ce lien vous étant exclusivement réservé, veuillez donc ne pas le transmettre à autrui.** Si vous connaissez des chefs de MSL qui n’ont pas reçu d’invitation, veuillez les encourager à communiquer avec nous pour recevoir un lien unique d’invitation au sondage.  Pour vous remercier de votre participation, nous vous ferons part de certains résultats avant la publication de l’ensemble des résultats du sondage.  Si vous avez des questions ou des préoccupations, n’hésitez pas à communiquer avec nous. Nous vous remercions à l’avance de votre contribution.  Cordialement,  **Lisa Cesario** au nom du **Réseau Canadien de MSL**  Directrice, Stratégies médicales  Affaires médicales et réglementation  Hoffmann-La Roche Limitée  7070 Mississauga Road  Mississauga (Ontario)  L5N 5M8 Canada  Cellulaire : 647-289-4489  Courriel : lisa.cesario@roche.com  Pour obtenir du soutien technique, envoyez un courriel à QuestionPro à l’adresse suivante : [ashok.singh@questionpro.com](mailto:ashok.singh@questionpro.com).  © 2020 Hoffmann-La Roche Ltd. All Rights Reserved.  [Unsubscribe](https://rochecanada.cmail20.com/t/r-u-shhttj-oyuqlkdkd-c/) |
| **FINAL-REMINDER EMAIL** |
| Date: Jul 6, 2020  Sent from: lisa.cesario@roche.com  Subject Line: MSL Survey is closing – share your insights now! / Clôture du sondage d’évaluation des MSL – Exprimez-vous maintenant!  Keywords: MSL metrics survey / Sondage d’évaluation des MSL    ----- Communication française à suivre ------  Dear <first name>    The Canadian Medical Science Liaison (MSL) Network survey of MSL leaders will be closing tomorrow. Don’t delay – share your insights today. In appreciation of your participation, we will share select results with you in advance of publishing the full study results.    The survey will take approximately 20 minutes to complete and is best viewed on a computer or tablet. The survey will be open until **July 7, 2020**. More information can be found on the first page of the survey prior to your consent.    “Take Survey Now” (image link)    **This link is unique to you, so please do not share it with others.**    If you have any questions or concerns, please do not hesitate to contact us. Thank you, in advance, for your contribution.    Sincerely,    **Lisa Cesario B.Sc.Phm** on behalf of **the Canadian MSL Network**  Medical Strategy Director  Medical and Regulatory Affairs  Hoffmann-La Roche Limited  7070 Mississauga Road  Mississauga, ON L5N 5M8 Canada  Mobile: 647-289-4489  Mail to: lisa.cesario@roche.com    For technical support, contact [ashok.singh@questionpro.com](mailto:ashok.singh@questionpro.com) at QuestionPro.  **---------**  Cher/Chère <first name>  Le sondage auprès des chefs des MSL du Réseau Canadien d’agents de liaison Médicoscientifique (MSL) se termine **demain**. N’attendez plus, exprimez-vous dès aujourd’hui. Pour vous remercier de votre participation, nous vous ferons part de certains résultats avant la publication de l’ensemble des résultats du sondage.  Le sondage prend environ 20 minutes à remplir et sera mieux consultée sur un ordinateur ou une tablette. Le sondage sera ouverte jusqu'au **7 juillet 2020**. Vos réponses sont anonymisées. De plus amples renseignements figurent sur la première page du sondage avant la section de consentement.   « Remplir le sondage maintenant » (lien visuel)  **Ce lien vous étant exclusivement réservé, veuillez donc ne pas le transmettre à autrui.**  Si vous avez des questions ou des préoccupations, n’hésitez pas à communiquer avec nous. Nous vous remercions à l’avance de votre contribution.  Cordialement,  **Lisa Cesario** au nom du **Réseau Canadien de MSL**  Directrice, Stratégies médicales  Affaires médicales et réglementation  Hoffmann-La Roche Limitée  7070 Mississauga Road  Mississauga (Ontario)  L5N 5M8 Canada  Cellulaire : 647-289-4489  Courriel : lisa.cesario@roche.com    Pour obtenir du soutien technique, envoyez un courriel à QuestionPro à l’adresse suivante : [ashok.singh@questionpro.com](mailto:ashok.singh@questionpro.com).  © 2020 Hoffmann-La Roche Ltd. All Rights Reserved.  [Unsubscribe](https://rochecanada.cmail20.com/t/r-u-shhttj-oyuqlkdkd-c/) |
